# Lymph node γδ and αβ CD8^+^ T cells share migratory properties

**DOI:** 10.1038/s41598-018-27339-8

**Published:** 2018-06-12

**Authors:** Milas Ugur, Anne Kaminski, Oliver Pabst

**Affiliations:** 1 Institute of Molecular Medicine, RWTH Aachen University, Aachen, Germany; 2 Present Address: Department of Microbiology and Immunology, The University of Melbourne, Melbourne, Australia

## Abstract

During immune responses, T cells differentiate into subsets with different functions and migratory properties. Here we characterize migratory behavior of endogenous αβ CD8^+^ and γδ T cells in lymph nodes by long-term tracking following *in vivo* photoconversion. We identified subsets of γδ T cells with distinct circulation kinetics that closely mirrored migratory subsets of αβ CD8^+^ T cells. Notably, αβ CD8^+^ and γδ T cells both comprised resident populations which stayed in lymph nodes for 4 weeks without circulation or proliferation. Furthermore, in contrast to the common conception, we observed that central memory αβ CD8^+^ T cells circulate with slower kinetics than naïve cells. Our results show that, similar to αβ T cells, γδ T cells can acquire distinct migratory properties during their development and differentiation and reveal unexpected intricacies of T cell migratory patterns.

## Introduction

T cell responses require effective T cell migration to infected tissues while maintaining sufficient immunosurveillance of uninfected tissues. This balance is achieved by different T cell subsets with particular migratory properties and circulation kinetics^1,2^. Naïve T cells continuously circulate through secondary lymphoid organs (SLOs) until they encounter their cognate antigens and differentiate into effector T cells that preferentially migrate into non-lymphoid tissues. After the effector phase, T cells can differentiate into classically defined memory subsets as central memory (T_CM_), which circulates between SLOs, effector memory (T_EM_), which circulates between spleen and non-lymphoid tissues and resident memory (T_RM_), which stays in non-lymphoid tissues without circulation.

Diversity in T cell migratory behavior is realized by specific combinations of chemokine receptors, integrins and selectins, as well as other homing factors. For example, both T_CM_ and naïve T cells express high levels of L-selectin (CD62L), CCR7 and S1PR1 which facilitates their circulation through lymph nodes (LNs)^1,2^. On the other hand, T_RM_ cells generally express low levels of these molecules, which contributes to their recruitment to and residency in non-lymphoid tissues^1,2^.

T cells of the vertebrate immune system can be divided into αβ and γδ T cells based on their T cell receptor (TCR) chains and αβ T cells are further classified as CD4^+^ helper and CD8^+^ cytotoxic T cells. Although γδ T cells represent only 1–2% of all T cells in LNs of human and mice, their frequency can be significantly higher in non-lymphoid tissues such as gut epithelium and skin epidermis^3–6^. Interestingly, γδ T cells expressing certain γ and/or δ chains are enriched in specific non-lymphoid organs, which is suggested to be due to specific retention and/or migration^3–8^. Most studies addressing migratory subsets of T cells focus on αβ T cells and less is known about circulation characteristics of γδ T cells. This is partially due to their low frequency in LNs, poorly understood differentiation pathways, heterogeneity in their TCR activation mechanisms and limitations of conventional experimental approaches^3–6^.

Recently, photoconversion-based cell tracking methods emerged as powerful tools to investigate T cell migration *in vivo*^9–13^. These methods employ photoconvertible proteins that change their fluorescence upon illumination with light at certain wavelengths^14^. Lymphocytes expressing photoconvertible proteins can be labelled in specific organs with local illumination and subsequently tracked *in vivo*. This allows spatiotemporal investigation of T cell circulation in LNs without manipulating entry or exit rates of T cells. However, regular protein turnover limits the use of this approach as photoconverted proteins within a cell are replaced by non-photoconverted proteins due to protein degradation and new protein synthesis. This results in rapid fading of photoconverted cells and none of the reported transgenic mouse models allows long-term *in vivo* tracking of T cells^10–12^. To overcome this limitation, we previously generated a histone-fused green-to-red photoconvertible protein (H2B-Dendra2) which dramatically increased the half-life of the native Dendra2 protein^15^. By using bone-marrow chimeras that express H2B-Dendra2, we identified resident populations of αβ CD4^+^ T cells in lymphoid organs^15^.

Here, we extend the long-term tracking of T cells to αβ CD8^+^ and γδ T cells using a transgenic mouse model that expresses a stabilized photoconvertible protein. We show that γδ T cells in LNs can be classified into subsets with different migratory characteristics that resemble those of αβ CD8^+^ T cells. Moreover, we identified resident populations of αβ CD8^+^ and γδ T cells in both skin and gut draining LNs that stayed in LNs without circulation or proliferation. Our results suggest that αβ CD4^+^ and CD8^+^ T cells as well as γδ T cells show highly congruent migratory patterns.

## Results

### γδ T cell subsets express different levels of migration-related genes

**Figure 1:**
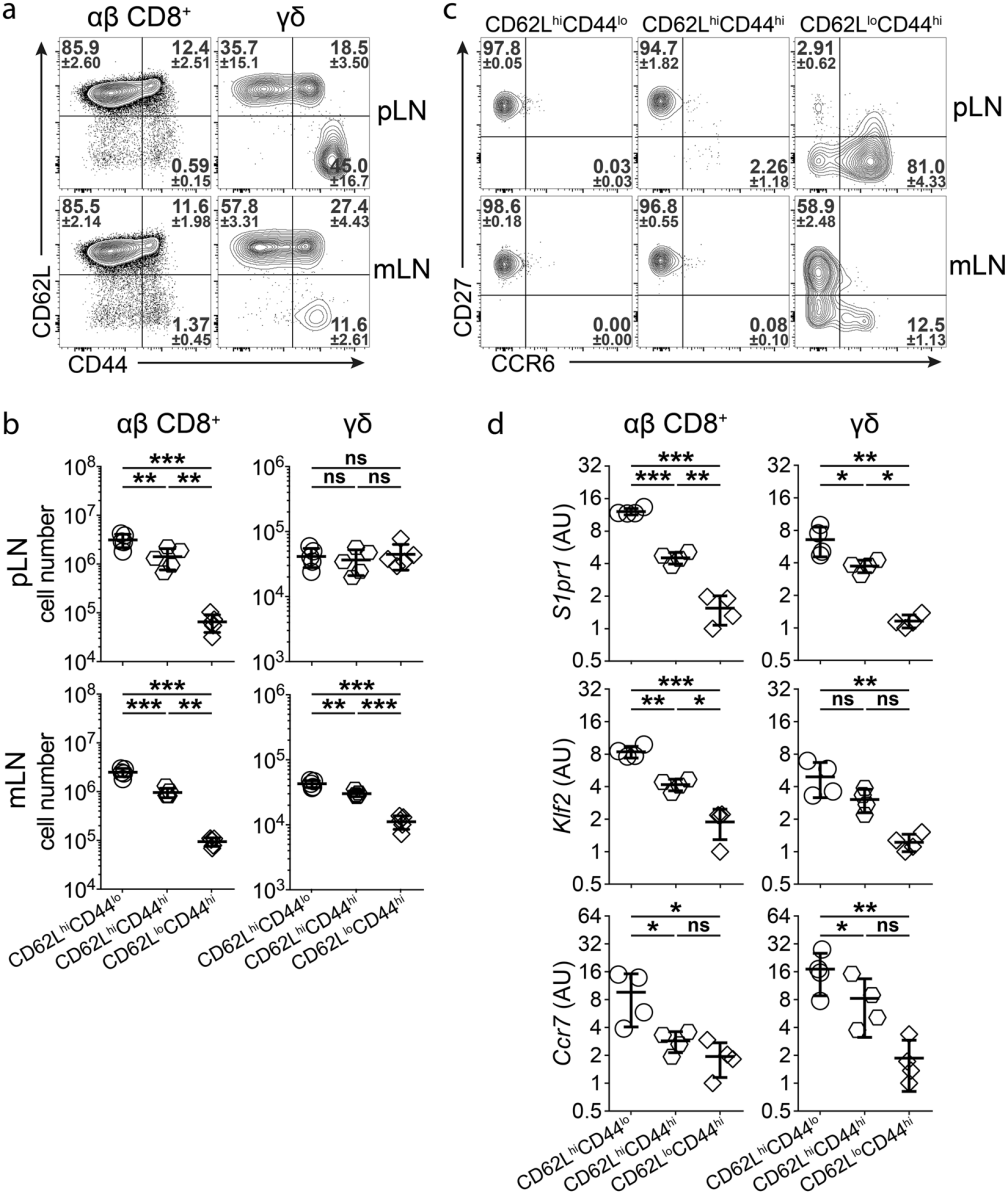
γδ T cells in LNs can be divided into distinct subsets using CD62L and CD44 expression. (**a**) CD62L and CD44 expression in αβ CD8^+^ and γδ T cells from skin-draining peripheral (top) and gut-draining mesenteric (bottom) LNs of untreated WT mice. Numbers show frequencies of respective gates (n = 6–9 mice in 5 independent experiments, mean ± SD). (**b**) Total cell numbers of CD62L^hi^CD44^lo^, CD62L^hi^CD44^hi^ and CD62L^lo^CD44^hi^ αβ CD8^+^ and γδ T cells from skin-draining peripheral (top) and gut-draining mesenteric (bottom) LNs of untreated WT mice (n = 5 mice in 2 independent experiments, mean ± SD, one-way ANOVA with Tukey’s multiple comparisons test, **P < 0.01; ***P < 0.001; ns: not significant). (**c**) CD27 and CCR6 expression in CD62L^hi^CD44^lo^, CD62L^hi^CD44^hi^ and CD62L^lo^CD44^hi^ γδ T cells from skin-draining peripheral (top) and gut-draining mesenteric (bottom) LNs of untreated WT mice. Numbers show frequencies of respective gates (n = 5 mice in 2 independent experiments, mean ± SD). (**d**) Expression levels of *S1pr1*, *Klf2*, *Ccr7* in CD62L^hi^CD44^lo^, CD62L^hi^CD44^hi^ and CD62L^lo^CD44^hi^ αβ CD8^+^ and γδ T cells from mLN of untreated WT mice (n = 4 pools of 10–12 mice in 4 independent experiments, mean ± SD, one-way ANOVA with Tukey’s multiple comparisons test, AU: arbitrary units, *P < 0.05; **P < 0.01; ***P < 0.001; ns: not significant).

CD62L and CD44 are commonly used to discriminate αβ T cells with different migratory properties in mice^1,2^. For αβ CD8^+^ T cells, the CD62L^lo^CD44^hi^ population contains T_EM_, T_RM_ and recently activated T cells whereas the CD62L^hi^CD44^hi^ and CD62L^hi^CD44^lo^ populations represent T_CM_ and naïve T cells, respectively. To explore the suitability of this classification to stratify populations of γδ T cells, we stained γδ T cells (CD19^−^CD3^+^TCRβ^−^TCRγδ^+^) from LNs of unmanipulated wild type mice for CD62L and CD44. Similar to αβ CD8^+^ T cells (CD19^−^CD3^+^TCRγδ^−^TCRβ^+^CD4^−^CD8^+^), we observed three major populations of γδ T cells (Fig. 1a,b). Frequency of CD62L^lo^CD44^hi^ γδ T cells was higher in skin-draining peripheral LNs (pLN) compared to gut-draining mesenteric LNs (mLN) and these cells expressed higher levels of CD44 in pLN, indicating site-specific accumulation of different subsets γδ T cells (Fig. 1a).

Populations of γδ T cells defined by expression of CD62L and CD44 differed from the commonly used classification into CD27^+^ IFNγ-producers and CCR6^+^ IL-17-producers^3–7^. CD62L^hi^CD44^lo^ and CD62L^hi^CD44^hi^ γδ T cells were almost exclusively CD27^+^CCR6^−^ in both pLN and mLN (Fig. 1b). In contrast, CD62L^lo^CD44^hi^ γδ T cells were mostly CCR6^+^ in pLN and mostly CD27^+^ in mLN (Fig. 1b), which is in accordance with a recently published study^16^. These results show that gating of γδ T cells using CD62L and CD44 provides information on γδ T cell diversity that is not apparent with CD27 and CCR6 staining.

Since both CD62L and CD44 have been established as informative markers to identify subsets of αβ T cells with distinct migratory behavior, we hypothesized that these markers might also divide γδ T cells into potentially distinct migratory subsets^1,2^. We thus further explored the migratory characteristics of these subpopulations of γδ T cells along with migratory subsets of αβ CD8^+^ T cells from the same organs.

Subsets of αβ CD8^+^ and γδ T cells from mLN of unmanipulated wild type mice were purified by cell sorting (Supplementary Fig. 1) and analyzed for expression of *S1pr1*, a central regulator of T cell egress from LNs, *Klf2*, a transcription factor regulating the expression of several T cell migration-related genes, and *Ccr7*, the dominant chemokine receptor for naïve T cell homing into LNs^17,18^. For both αβ CD8^+^ and γδ T cells, subsets defined by CD62L and CD44 expression showed differential expression of *S1pr1*, *Klf2* and *Ccr7* (Fig. 1d). On average, CD62L^hi^CD44^lo^ cells expressed the highest levels of these genes while CD62L^lo^CD44^hi^ expressed the lowest and CD62L^hi^CD44^hi^ cells expressed intermediate levels (Fig. 1d). These results demonstrate the heterogeneity among γδ T cells with respect to expression of migration-related genes and support the concept of defining migratory subsets of γδ T cells similar to αβ T cells based on CD62L and CD44 expression.

### H2B-Dendra2 protein allows long term tracking of lymphocytes

**Figure 2:**
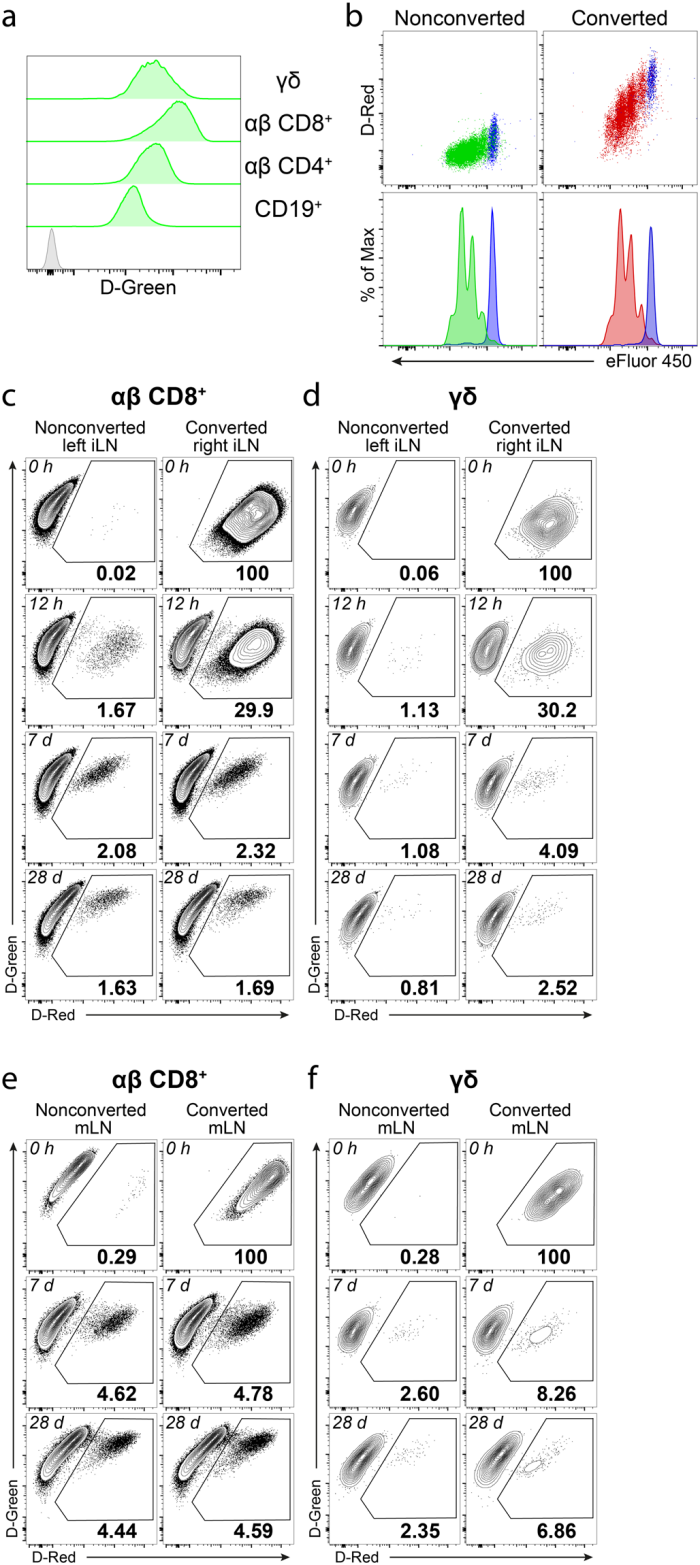
Vav-H2B-Dendra2 (VHD) transgenic mice allow tracking of lymphocytes for at least 28 days after photoconversion. (**a**) Expression of H2B-Dendra2 protein (D-Green) in CD19^+^ B cells, αβ CD4^+^ T cells, αβ CD8^+^ T cells and γδ T cells from a VHD mouse (green histograms) and αβ CD8^+^ T cells from a WT mouse (gray histogram) (representative of >50 mice in >10 experiments). (**b**) *In vitro* proliferation of *ex vivo* photoconverted (right) and nonconverted (left) αβ CD8^+^ T cells from VHD mice that are labelled with eFluor 450 proliferation dye. Blue histograms show unstimulated cells (representative of 4 experiments). (**c** and **d**) Frequency of D-Red^+^ cells among αβ CD8^+^ (**c**) and γδ (**d**) T cells immediately, 12 hours, 7 days or 28 days after photoconversion of inguinal LN in VHD mice (representative of ≥2 mice in ≥2 independent experiments). (e and f) Frequency of D-Red^+^ cells among αβ CD8^+^ (**e**) and γδ (**f**) T cells immediately, 7 days or 28 days after photoconversion of mesenteric LN in VHD mice (representative of ≥2 mice in ≥2 independent experiments).

Antigen experienced αβ CD8^+^ T cells and γδ T cells constitute only a minor population of T cells in LNs. Moreover, development and differentiation of these endogenous T cell populations can occur before birth or in response to undefined antigens. To study circulation kinetics of endogenous T cell populations, we generated a transgenic mouse that expresses a fusion protein of histone H2B and the photoconvertible Dendra2 protein (H2B-Dendra2) under the control of the hematopoietic Vav promoter^15,19^. T and B cells from Vav-H2B-Dendra2 (VHD) mice expressed high levels of H2B-Dendra2 protein (Fig. 2a). Exposure of T cells from VHD mice to violet light caused irreversible photoconversion of native H2B-Dendra2 (D-Green) protein into photoconverted H2B-Dendra2 (D-Red) protein (Fig. 2c–f). In VHD mice, photoconverted D-Red^+^ T cells could reliably be detected at least 4 weeks after photoconversion of LNs (Fig. 2c–f). This indicates that histone fusion of Dendra2 provides a considerable improvement over previously reported transgenic mice expressing photoconvertible proteins in particular to enable long-term tracking of photoconverted cells *in vivo*^10–12^.

Proliferating T cells lost the D-Red signal as cells divide and the photoconverted Dendra2 proteins are distributed to daughter cells. D-Red^+^ T cells that proliferate after photoconversion lost detectable levels of the D-Red signal after 2–3 divisions (Fig. 2b). Thus, D-Red signal intensity after photoconversion also specifies the proliferation history of photoconverted cells.

In published work, photoconversion of LNs was performed on LNs exposed by surgery, a step required for efficient photoconversion and labelling of all lymphocytes in the LN^11,15^. However, surgery inevitably alters immune homeostasis and might affect migration and function of immune cells in the draining LN. To avoid such potential issues, we established an approach to photoconvert all T cells in inguinal LN (iLN) through the skin without surgical exposure. Photoconversion of iLN through the skin efficiently marked virtually all T cells, including γδ T cells (Fig. 2c,d). After photoconversion of iLN through the skin, we did not observe any differences between photoconverted and contralateral nonconverted iLNs in terms of cell numbers or dendritic cell frequencies, suggesting that the procedure did not cause any major disturbance of immune homeostasis (Supplementary Fig. 3). In conclusion, we propose that the VHD mouse provides a powerful model to study T cell circulation and migration, especially for long term studies in skin-draining LNs.

### αβ CD8^+^ and γδ T cells in lymph nodes contain subsets with different turnover rates

To study circulation kinetics of γδ T cell subsets, we photoconverted one iLN of VHD mice and analyzed the frequency of D-Red^+^ cells remaining in the converted iLN over 24 hours. After photoconversion, D-Red^+^ T cells in the converted iLN may leave the LN over time via lymphatics and enter blood, while D-Red^−^ T cells home to the converted iLN to replace them. This process results in a gradual decrease in the frequency of D-Red^+^ cells in the photoconverted LN and builds a circulating population of D-Red^+^ T cells. This circulating population of D-Red^+^ T cells can home back to the photoconverted iLN as well as to other nonconverted LNs. To determine effective turnover rates in the photoconverted iLN, we subtracted the frequency of D-Red^+^ cells in the nonconverted contralateral iLN of the same mouse for each subset to account for the recirculating D-Red^+^ cells, which was usually in the range of 1–2%.

**Figure 3:**
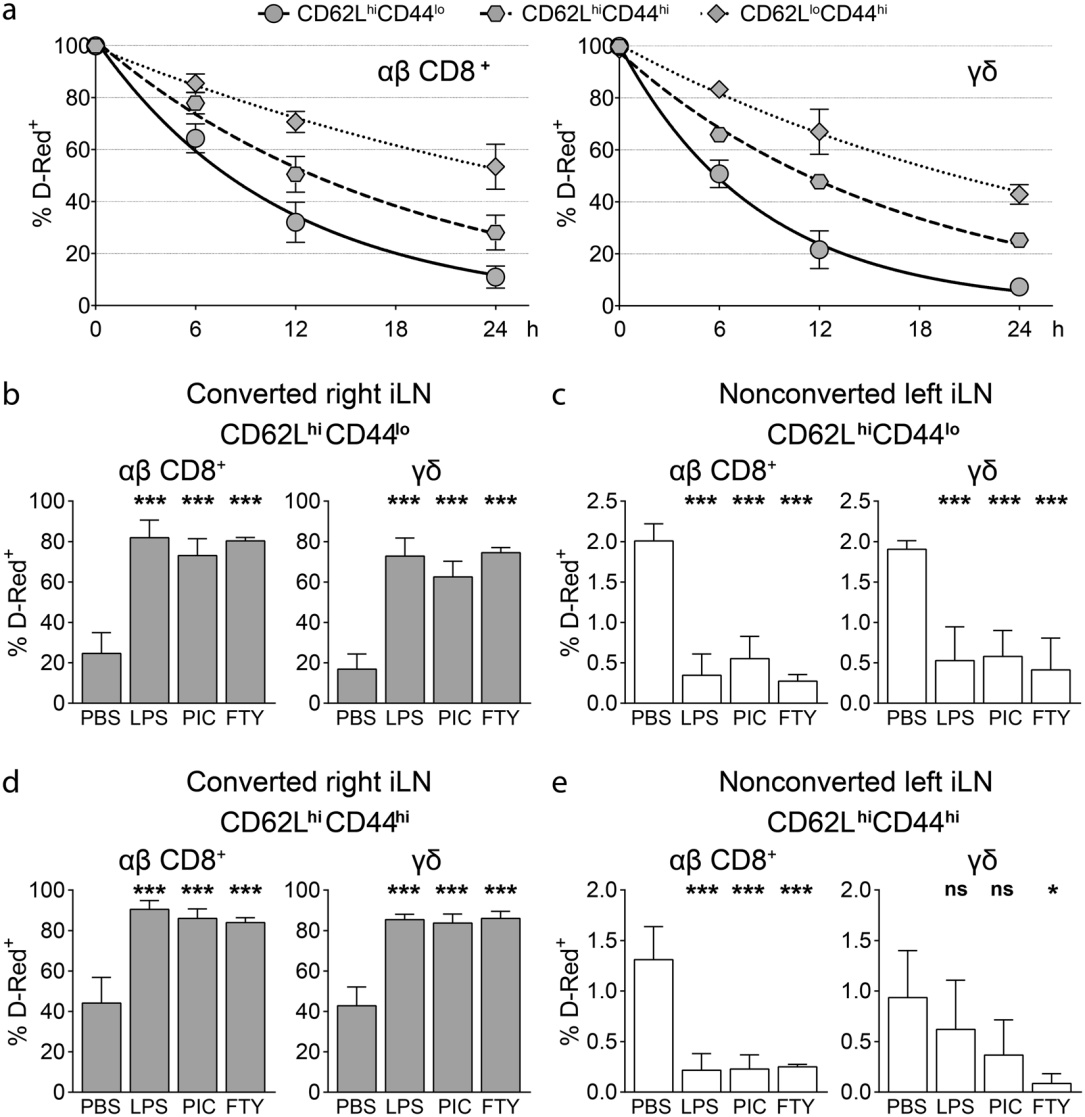
CD62L^hi^CD44^lo^, CD62L^hi^CD44^hi^ and CD62L^lo^CD44^hi^ αβ CD8^+^ and γδ T cells have different short term circulation kinetics. (**a**) Corrected frequency of D-Red^+^ cells among CD62L^hi^CD44^lo^, CD62L^hi^CD44^hi^ and CD62L^lo^CD44^hi^ αβ CD8^+^ T cells (left) and γδ T cells (right) in the photoconverted iLN at the indicated time points after photoconversion. Correction is performed by subtracting the frequency of D-Red^+^ cells in the nonconverted iLN from the frequency of D-Red^+^ cells in the converted iLN for the respective population at each time point (n = 2–4 mice per time point in 3 independent experiments, mean ± SD, one phase exponential decay curves). (**b**–**e**) VHD mice are injected intraperitoneally with either PBS, LPS, poly(I:C) (PIC) or FTY720 (FTY) 2 hours after photoconversion of iLN and analyzed 16 hours after photoconversion. (**b** and **c**) Frequency of D-Red^+^ cells among CD62L^hi^CD44^lo^ among αβ CD8^+^ T cells (left) and γδ T cells (right) in photoconverted (**b**) and nonconverted (**c**) iLNs (n = 3–5 mice per group in 4 independent experiments, mean ± SD, one-way ANOVA with Tukey’s multiple comparisons test, ***P < 0.001) (**d** and **e**) Frequency of D-Red^+^ cells among CD62L^hi^CD44^hi^ among αβ CD8^+^ T cells (left) and γδ T cells (right) in photoconverted (**d**) and nonconverted (**e**) iLNs (n = 3–5 mice per group in 4 independent experiments, mean ± SD, one-way ANOVA with Tukey’s multiple comparisons test, *P < 0.05; ***P < 0.001; ns: not significant).

Subpopulations of T cells as defined by CD62L and CD44 expression varied substantially in their circulation kinetics. However, for corresponding subpopulations defined by these markers, we observed a striking similarity in the turnover rates of αβ CD8^+^ and γδ T cells (Fig. 3a, Supplementary Fig. 4). For both αβ CD8^+^ and γδ T cells, frequency of D-Red^+^ cells decreased fastest for the CD62L^hi^CD44^lo^ subset and slowest for the CD62L^lo^CD44^hi^ subset (Fig. 3a, Supplementary Fig. 4). αβ CD8^+^ CD62L^hi^CD44^lo^ T cells showed a half-life (t_1/2_) of 7.7 hours, which was in a similar range with γδ CD62L^hi^CD44^lo^ cells. For both of these populations, ∼90% of the cells were replaced in the photoconverted LN within 24 hours, which is consistent with previous studies^11,13^. Surprisingly, the CD62L^hi^CD44^hi^ subset of αβ CD8^+^ T cells had an intermediate turnover rate (t_1/2_ = 12.8 hours), indicating that αβ CD8^+^ T_CM_ have a slower turnover rate than naïve cells in LNs (Fig. 3a, Supplementary Fig. 4). A similar difference between CD62L^hi^CD44^lo^ (t_1/2_ = 5.8 hours) and CD62L^hi^CD44^hi^ (t_1/2_ = 11.7 hours) subsets was also observed for γδ T cells, further emphasizing the similarities between circulation kinetics of αβ CD8^+^ and γδ T cell subsets (Fig. 3a, Supplementary Fig. 4). High expression of *S1pr1*, *Klf2* and *Ccr7* largely correlated with faster turnover rates, underlining the importance of these genes in the regulation of T cell circulation through LNs (Figs 1d and 3a). In conclusion, CD62L and CD44 expression distinguishes subpopulations of γδ T cells that differ in their short-term circulation kinetics and these subsets resemble their corresponding αβ CD8^+^ counterparts.

Inflammation and infection affect αβ T cell circulation by changing the rates of T cell entry into SLOs and/or egress from SLOs^9,20–23^. To investigate the effects of systemic inflammation on γδ T cell circulation, we treated VHD mice with TLR agonist LPS and poly(I:C) or the egress blocking drug FTY720 two hours after photoconversion of the iLN. Photoconverted iLNs were analyzed 16 hours after photoconversion for frequencies of D-Red^+^ cells among CD62L^hi^CD44^lo^ and CD62L^hi^CD44^hi^ subsets of αβ CD8^+^ and γδ T cells. Expectedly, we observed a dramatic increase in the frequency of D-Red^+^ cells in both subsets of αβ CD8^+^ T cells in LPS, poly(I:C) or FTY720 treated mice compared to PBS treated controls (Fig. 3b,d). This increase was paralleled by a decrease in D-Red^+^ cell frequencies in the nonconverted contralateral iLN suggesting that fewer D-Red^+^ cells egressed from the photoconverted iLN (Fig. 3c,e). Notably, we observed an almost identical pattern for the changes in circulation kinetics of γδ T cells. The frequency of D-Red^+^ cells among γδ T cells strikingly increased in the photoconverted iLN after LPS, poly(I:C) and FTY720 treatments for all subsets (Fig. 3b,d, Supplementary Fig. 5). These results suggest that in addition to subset-specific turnover rates, mechanisms regulating changes in circulation kinetics during inflammation are also shared between αβ CD8^+^ and γδ T cells.

### Lymph nodes harbor resident αβ CD8^+^ and γδ T cells

**Figure 4:**
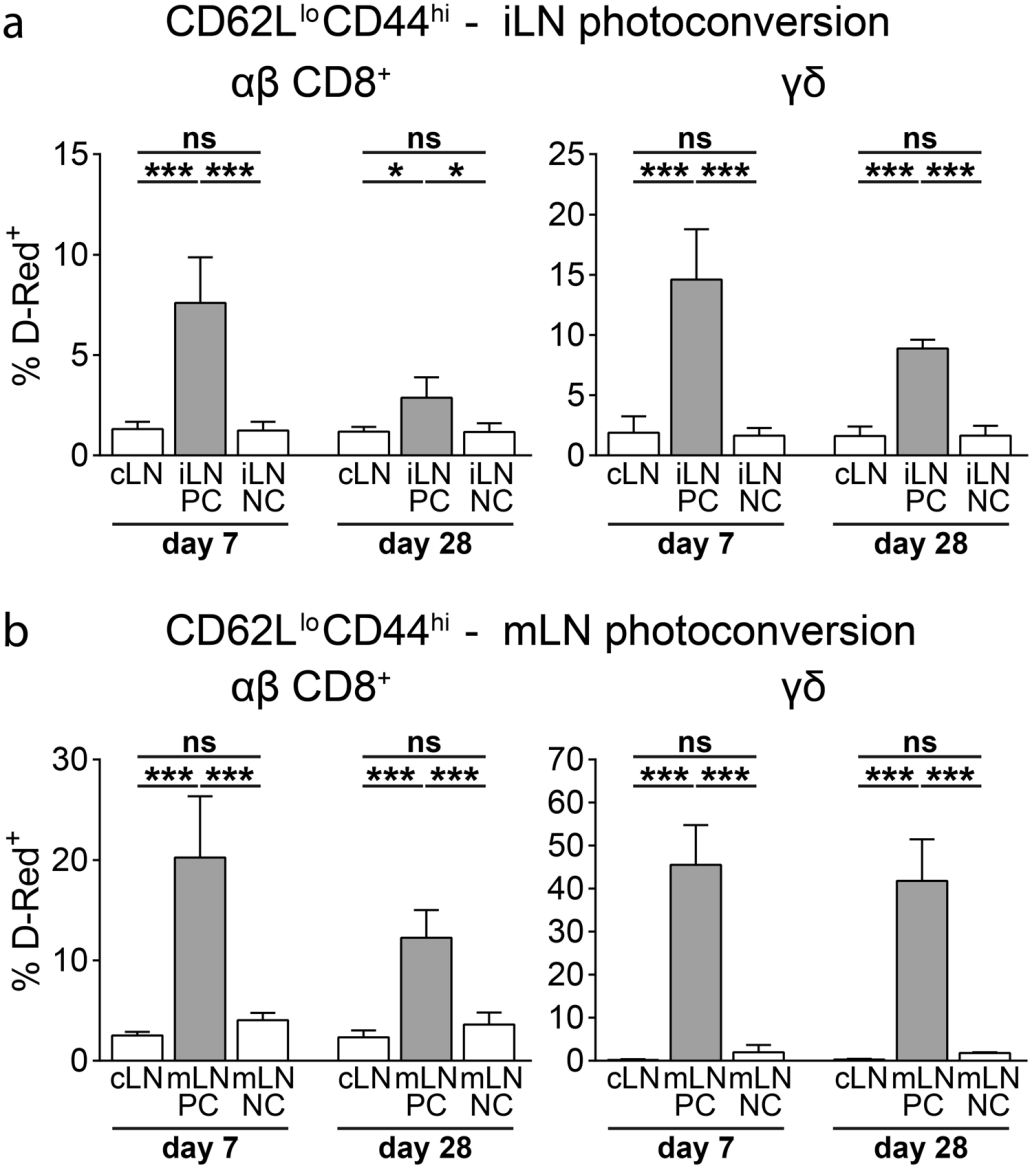
Peripheral and mesenteric LNs harbor resident γδ T cells. (**a**) Frequency of D-Red^+^ cells among CD62L^lo^CD44^hi^ among αβ CD8^+^ T cells (left) and γδ T cells (right) in the indicated LNs 7 and 28 days after photoconversion of iLN (n = 4–6 mice per time point in 5 independent experiments, mean ± SD, one-way ANOVA with Tukey’s multiple comparisons test, *P < 0.05; ***P < 0.001; ns: not significant). (**b**) Frequency of D-Red^+^ cells among CD62L^lo^CD44^hi^ for αβ CD8^+^ T cells (left) and γδ T cells (right) in the indicated LNs 7 and 28 days after photoconversion of mLN (n = 4–5 mice per time point in 5 independent experiments, mean ± SD, one-way ANOVA with Tukey’s multiple comparisons test, ***P < 0.001; ns: not significant).

Since we observed a relatively slow turnover rate for CD62L^lo^CD44^hi^ subsets of both αβ CD8^+^ and γδ T cells in our short-term analysis (24 hours or earlier after photoconversion), we performed a long-term analysis (7 days or later after photoconversion) to better describe the circulation kinetics of these populations. Similar to our short-term experiments, we photoconverted skin-draining iLN and gut-draining mLN of VHD mice and analyzed the frequency of D-Red^+^ cells in photoconverted and nonconverted LNs. 28 days after photoconversion of iLN or mLN, both CD62L^hi^CD44^lo^ and CD62L^hi^CD44^hi^ subsets of αβ CD8^+^ and γδ T cells largely equilibrated between photoconverted and nonconverted LNs (Supplementary Fig. 6). In contrast, the frequency of D-Red^+^ cells among both CD62L^lo^CD44^hi^ αβ CD8^+^ and γδ T cells in converted LNs was significantly higher compared to nonconverted LNs, suggesting the presence of LN-resident populations within these subsets (Fig. 4). Resident cells constituted up to 40% of all CD62L^lo^CD44^hi^ αβ CD8^+^ and γδ T cells, which identifies them as a substantial fraction of CD62L^lo^CD44^hi^ T cells in LNs. Frequency of LN-resident cells among the CD62L^lo^CD44^hi^ subset was higher among γδ T cells than αβ CD8^+^ T cells in both iLN and mLN although mLN generally contained higher frequencies of resident cells as compared to iLN (Fig. 4). Comparing the frequencies of resident cells between day 7 and day 28 after photoconversion, we observed an approximately 5% decrease in both iLN and mLN (Fig. 4). This decline might be due to several processes such as homeostatic proliferation, generation of new effector/memory cells, cell death or very slow circulation rate of these populations. In conclusion, LNs contain endogenous resident αβ CD8^+^ and γδ T cells under homeostatic conditions and these cells can make up a major proportion of CD62L^lo^CD44^hi^ cells.

## Discussion

Here, we describe circulation kinetics of endogenous αβ CD8^+^ and γδ T cells in LNs during steady state and systemic inflammation. Our observations are based on a novel transgenic VHD mouse model that enables both short-term (24 hours or earlier after photoconversion) and long-term (7 days or later after photoconversion) analysis of T cell circulation via hematopoietic expression of a histone-fused irreversibly photoconvertible protein. In contrast to previous approaches^10–13^, our model shows long term stability of the photoconverted protein which allows *in vivo* tracking of T cells for several weeks after *in situ* labeling. Furthermore, VHD mouse enables efficient photoconversion of all T cells in skin-draining LNs without the need for surgical exposure which was not achieved in a previous study^24^. Since reliable photoconversion of all T cells in a LN is essential for analysis of turnover rates, VHD mouse provides a valuable tool for the study of T cell circulation.

αβ CD8^+^ and γδ T cells have been suggested to migrate at similar speeds and patterns within LNs but different migratory subsets were not considered in this study^25^. CD62L and CD44 are commonly used to differentiate migratory populations of αβ T cells^1,2^. Both CD62L and CD44 have also been used to determine activation/differentiation status of γδ T cells^26–29^. Here we speculated that the surface markers CD62L and CD44 might identify subsets of γδ T cells with different migratory properties. Indeed, gating based on CD62L and CD44 expression identified γδ T cell subsets with different circulation kinetics and expression levels of migration-related genes that closely mimicked αβ CD8^+^ T cell subsets. These results imply the existence of generic migratory roles for T cells which can be assigned to both αβ and γδ T cells.

Naïve and central memory αβ CD8^+^ T cells are generally thought to share similar migration patterns^1,2^. Yet, our observations revealed important differences between these two subsets in terms of both turnover rates and expression of migration-related genes. T_CM_ cells are characterized by their expression of CCR7 and CD62L, which are important molecules for LN homing and also expressed by naïve T cells. However, LN homing of T_CM_ cells is less stringently dependent on CCR7 as compared to naïve cells and high surface CD62L expression is not a deterministic marker for T_CM_ differentiation^30–32^. During vaccinia and influenza infections, CD62L re-expression by activated T cells has been shown to be required for their homing into peripheral tissues^33^. Moreover, T_CM_ cells have been recently shown to migrate into non-lymphoid tissues via expression of core 2 O-glycans which generates functional ligands for E- and P-selectins^34^. A new subset of CX3CR1int peripheral memory T cells which can migrate into both LNs and peripheral tissues have also been described challenging the notion of TCM cells as being the only memory subset that can migrate into LNs^35^. In aggregate, these observations suggest that migratory properties of T_CM_ cells cannot be sufficiently described by the traditional T cell migration paradigm that unifies migratory behavior of T_CM_ and naïve T cells.

Central memory populations for human Vγ9^+^Vδ2^+^ T cells have also been proposed based on expression of CD45RA, CD45RO and CD27^36^. Our findings suggest the presence of a CD62L^hi^CD44^hi^ central memory-like subset of γδ T cells which showed striking similarities to αβ CD8^+^ T_CM_ cells based on circulation kinetics and expression of migration-related genes. However, further studies are needed to delineate whether this population also shows similarities to αβ T_CM_ cells in terms of memory potential, cytokine production and proliferative capacity.

Similar to αβ T cells, γδ T cells have been shown to require *S1pr1* and *Klf2* expression to leave the thymus and populate LNs in the periphery^37^. Furthermore, FTY720, a functional antagonist of S1PR1, blocked the egress of γδ T cells from LNs under homeostatic conditions and after imiquimod treatment^38,39^. Our results confirm these observations and indicate that both CD62L^hi^CD44^lo^ and CD62L^hi^CD44^hi^ γδ T cells depend on S1PR1 to egress from LNs. Interestingly, systemic inflammation also blocked egress of both CD62L^hi^CD44^lo^ and CD62L^hi^CD44^hi^ γδ T cells, almost as efficiently as FTY720 treatment. The physiological significance of this observation is still unknown. For αβ T cells, it has been suggested that early restriction of T cell egress might increase the frequency of naïve precursors in a LN before TCR-mediated S1PR1 downregulation in activated T cells^18,40^. Reduced egress of γδ T cells during inflammation may serve a similar function in terms of TCR diversity. Moreover, since γδ T cells are early producers of cytokines such as IL-17 and IFNγ in several infection models, increased retention of γδ T cells in a LN might also contribute to rapid innate responses against infections^3–6,16^.

Although initially T_RM_ populations have been described for non-lymphoid tissues, resident populations of effector/memory T cells in LNs have also been shown for αβ CD8^+^^41^, αβ CD4^+^^15,42–45^ and γδ^16,46,47^ T cells using either TCR transgenic or endogenous polyclonal T cells. Here we report the frequency of resident cells among endogenous polyclonal αβ CD8^+^ and γδ T cells in skin and gut-draining LNs under homeostatic conditions. Similar to other *in vivo* cell tracking approaches based on photoconversion, VHD mouse provides a proliferation sensitive labelling system, since the amount of photoconverted D-Red protein is halved with each cell division after photoconversion. Therefore, frequencies we observed for resident cells in LNs are probably underestimates due to possible homeostatic proliferation of these cells.

We observed higher frequencies of resident cells for both αβ CD8^+^ and γδ T cells in gut-draining mLN compared to skin-draining iLN, a pattern we also observed for resident αβ CD4^+^ T cells in our previous work^15^. This difference can be due to several factors such as priming history of a LN, type and maturation status of antigen presenting cells, stroma cell-derived factors and basal inflammatory tone in the respective compartment. Indeed, different adjuvants have been shown to have varying efficiencies in generating LN resident follicular helper T cells^48^.

Although little is known about the functional contributions of LN resident αβ T cells, they are presumed to support secondary responses. Due to their localization around subcapsular sinus and lymphatics within the LN, LN resident αβ CD8^+^ T cells are proposed to provide an early line of defense, similar to T_RM_ cells in non-lymphoid tissues^41^. Interestingly, LN resident IL-17-producing γδ T cells were also found in close proximity of subcapsular sinus^16,46^. LN resident memory γδ T cells were also found in interfollicular areas after *Listeria monocytogenes* infection and these cells clustered around myeloid cells after secondary infection^47^. Considering the diverse innate and adaptive characteristics of γδ T cells, it is conceivable to assume LN resident γδ T cells contributing to both primary and secondary immune responses.

Our results provide a comprehensive analysis of αβ CD8^+^ and γδ T cell circulation kinetics in LNs under homeostatic and inflammatory conditions. We identified distinct migratory subsets of γδ T cells which closely resembled those of αβ CD8^+^ T cells. Since migratory subsets of αβ CD8^+^ T cells are known to have distinct functional characteristics, we speculate that migratory subsets of γδ T cells might also have distinct contributions to immune responses.

## Methods

### Mice

C57BL6/J and Vav-H2B-Dendra2 (VHD) (B6.tg(HD2)) mice were bred and reared at the Animal Facility of RWTH Aachen University under specified pathogen free conditions. All mice used in the experiments were 6–12 weeks old. In some experiments, mice were injected intraperitoneally with LPS (20 µg per mouse, Sigma), poly(I:C) (100 µg per mouse, InvivoGen), FTY720 (25 µg per mouse, Sigma) or phosphate buffered saline (PBS) solution. Peripheral LN pools contained left and right inguinal, axillary and brachial LNs. All experiments were approved by North Rhein-Westphalia State Agency for Nature, Environment and Consumer Protection (Landesamt fur Natur, Umwelt und Verbraucherschutz Nordrhein-Westfalen, LANUV) and performed in accordance with relevant local guidelines and regulations. All experiments were performed in compliance with ethical regulations of German Law for the Protection of Animal Welfare (Tierschutzgesetz).

For the generation of VHD mice, H2B-Dendra2 coding sequence^9^ was cloned into HS21/45 *vav*-hCD4 plasmid^19^ with SfiI and NotI digestion, replacing the hCD4 coding sequence (Supplementary Fig. 2). HS21/45 *vav*-hCD4 plasmid contains the promoter elements of the vav promoter and these elements are denoted as HS 2, 1, 4 and 5 indicating DNAse hypersensitive regions around exon 1 of the vav gene. The resulting HS-21/45 *vav-*H2B-Dendra2 construct was linearized and injected into BDF1 fertilized eggs. After screening, mice expressing Dendra2 were backcrossed with C57BL/6J mice for at least 5 generations.

### Flow cytometry

Single cell suspensions were prepared by meshing LNs through nylon filters in PBS solution containing 3% fetal calf serum (PBS/FCS). Subsequent steps of surface staining were performed in PBS/FCS. Blocking was performed with PBS/FCS solution containing 5% rat serum. Appropriate combinations and concentrations of antibodies CD3 (17A2), TCRβ (H57-597), TCRγδ (GL3), CD62L (MEL-14), CD44 (IM7), CD8α (53-6.7), CD4 (RM4-5), CD19 (6D5), CD27 (LG.3A10), CCR6 (FAB590P), B220 (RA3-6B2), CD11c (N418), MHCII (M5/114.15.2), CD11b (M1/70), CD64 (X54-5/7.1), and appropriate isotype control antibodies were used for staining. Antibodies were bought from BioLegend, eBioscience, BD or R&D. All measurements were done on LSR Fortessa (BD) and analyzed using FlowJo software (Tree Star). Cell sorting was performed on Aria IIu (BD).

### *In vitro* T cell activation

T cells were isolated from LNs by meshing through nylon filters and labeled with Cell Proliferation Dye eFlour 450 (eBioscience) according to manufacturer’s instructions. For the stimulation, T cells were incubated in full RPMI medium supplemented with IL-2 and Dynabeads Mouse T-Activator CD3/CD28 beads (Invitrogen) according to manufacturer’s instructions.

### Real-time PCR

RNA isolation from sorted populations was performed with RNeasy Micro kit (QIAGEN) followed by cDNA synthesis with SuperScript III Reverse Transcriptase using random hexamer primers. Real-time PCR was performed using a CFX96 Real Time System (BioRad) and SYBR Green detection (Takara) with primers; *Gapdh*-forward 5′-GTGCCAGCCTCGTCCCGTAG-3′, *Gapdh*-reverse 5′-TTGCCGTGAGTGGAGTCATAC-3′, *S1pr1*-forward 5′-GGAGGTTAAAGCTCTCCGC-3′, *S1pr1*-reverse 5′-CGCCCCGATGTTCAAC-3′, *Klf2*-forward 5′-ACCAACTGCGGCAAGACCTA-3′, *Klf2*-reverse 5′-CATCCTTCCCAGTTGCAATGA-3′, *Ccr7*-forward 5′-TGTACGAGTCGGTGTGCTTC-3′, *Ccr7*-reverse 5′-GGTAGGTATCCGTCATGGTCTTG-3′. All expression levels were normalized to *Gapdh* expression and relative expression levels were determined using 2^−ΔΔCt^ method.

### Photoconversion

Photoconversion of LNs was performed as previously described with some modifications^15^. Briefly, mice were anaesthetized using ketamine and xylazine. For photoconversion of mLN, small intestine and cecum were exposed through a small midline incision into the skin and the abdominal wall. Each mLN was illuminated for 20 seconds. After surgery, all wounds were closed with suture and metal clips. For photoconversion of iLN, hair in both right and left flank was gently shaved with a hair clipper without using depilating cream and gently cleaned with PBS. The right iLN was located using blood vessels as references and illuminated for 30–60 seconds through the skin.

### Statistics

Statistical analysis was performed using GraphPad Prism software. For comparisons, paired Student’s t-test or one-way ANOVA with Tukey’s multiple comparisons test were used as described in the figure legends. In Fig. 3a, one phase exponential decay curves are calculated by least squares (ordinary) fit with K > 0 and plateau is constant and equal to 0. Data are presented as mean ± SD (standard deviation). P < 0.05 are considered as significant. In all figures; *P < 0.05, **P < 0.01, ***P < 0.001, ns: not significant.

## Electronic supplementary material

Supplementary Figures S1 - S6
