# Supplementary material for: Lymph node γδ and αβ CD8+ T cells share migratory properties
Source: Sci Rep. 2018 Jun 12;8:8986. doi: 10.1038/s41598-018-27339-8 (PMC5997669; doi:10.1038/s41598-018-27339-8)
Supplement: Supplementary file 1 — Supplementary Figures S1 - S6 [file 41598_2018_27339_MOESM1_ESM.pdf]

Lymph node γδ and αβ CD8^{+} T cells share migratory properties

Milas Ugur[1, 2], Anne Kaminski[1], Oliver Pabst[1*]

[1] Institute of Molecular Medicine, RWTH Aachen University, Aachen, Germany

[2] Current address: Department of Microbiology and Immunology, The University of Melbourne, Melbourne, Australia

Supplementary Figure S1

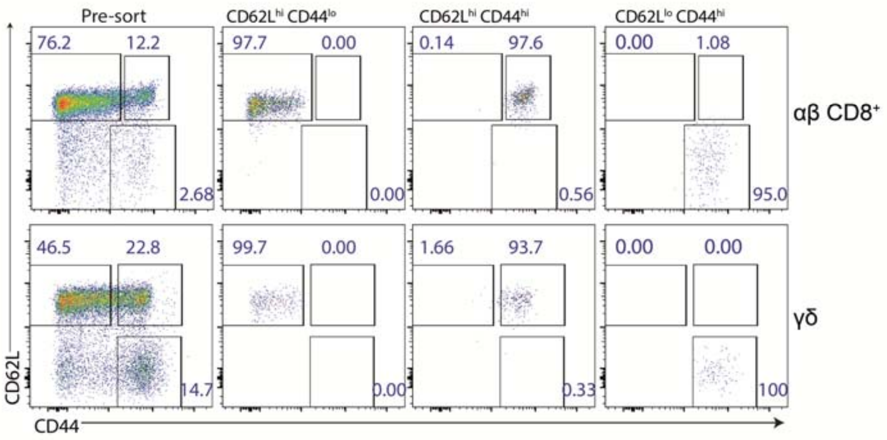

Figure S1 | Sorting of CD62L^{hi}CD44^{lo}, CD62L^{hi}CD44^{hi} and CD62L^{lo}CD44^{hi} αβ CD8^{+} (top) and γδ (bottom) T cells for gene expression analysis. CD62L and CD44 expression among T cells before (left-most column) and after sorting (representative of 4 independent experiments). T cells were sorted from mLN of untreated WT mice. αβ CD8^{+} T cells were defined as DAPI^{-}CD19^{-}CD3^{+}TCRγδ^{-}TCRβ^{+}CD4^{-} CD8^{+} cells and γδ T cells were defined as DAPI^{-}CD19^{-}CD3^{+}TCRβ^{-}TCRγδ^{+} cells. **Supplementary Figure S2**

Supplementary Figure S2

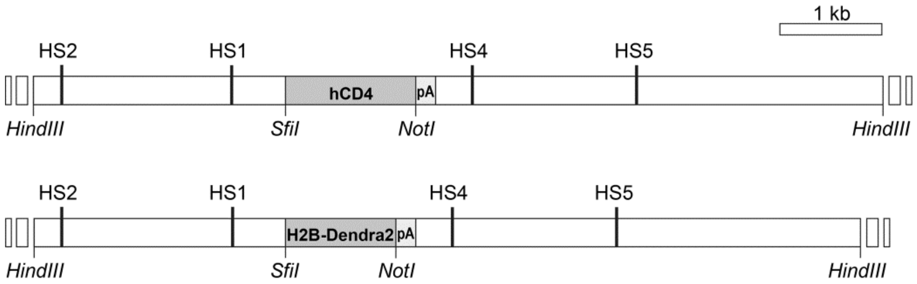

Figure S2 | Structure of the DNA construct used for the generation of the VHD mouse. Diagrams showing the restriction sites, DNase hypersensitive sites (HS) around exon 1 of the vav gene and polyadenylation sites (pA) on HS21/45 *vav*-hCD4 (top, Ogilvy et al., 1999) and HS21/45 *vav*-H2B-Dendra2 (bottom, this paper) plasmids. For the generation of the VHD mouse line, HS21/45 *vav*-H2B-Dendra2 plasmid was linearized with HindIII digestion and injected into BDF1 fertilized eggs.

Supplementary Figure S3

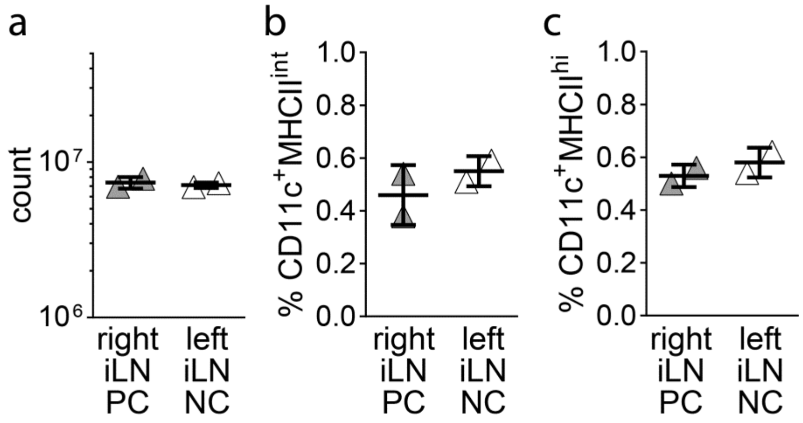

Figure S3 | Photoconversion of inguinal lymph node through the skin does not affect total cell numbers or dendritic cell frequencies in the photoconverted lymph node. (**a**-**c**) Right inguinal lymph nodes of WT mice were photoconverted through the skin and the mice are analyzed 24 hours after photoconversion. Total cell numbers (**a**), frequency of CD11c$^+$MHCII^{int} resident dendritic cells among all cells (**b**) and frequency of CD11c$^+$MHCII^{hi} resident dendritic cells among all cells (**c**) are shown (n = 2 mice in 1 experiment, mean±SD,).

Supplementary Figure S4

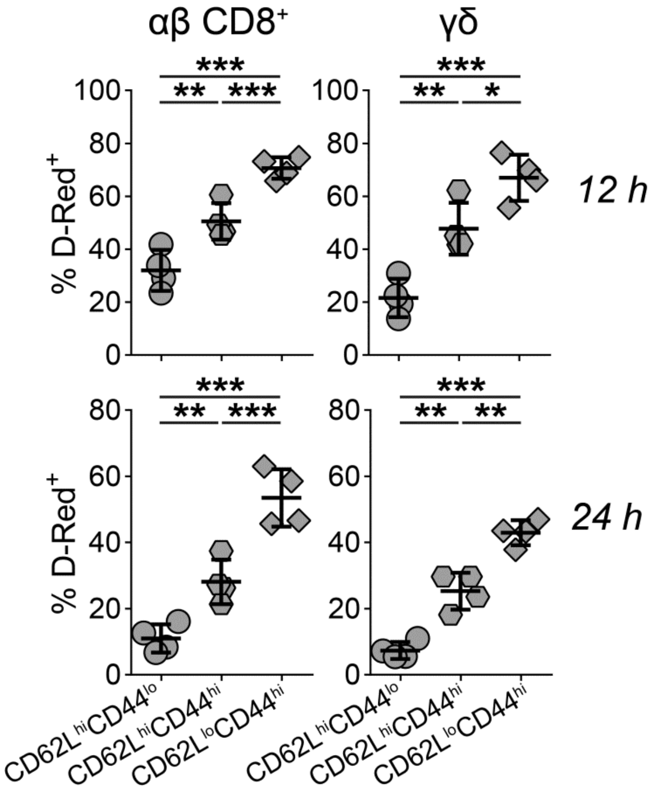

Figure S4 | CD62L^{hi}CD44^{lo}, CD62L^{hi}CD44^{hi} and CD62L^{lo}CD44^{hi} αβ CD8^{+} and γδ T cells have different short term circulation kinetics. Statistical analysis of data from **Figure 3a**. Corrected frequency of D-Red^{+} cells among CD62L^{hi}CD44^{lo}, CD62L^{hi}CD44^{hi} and CD62L^{lo}CD44^{hi} αβ CD8^{+} T cells (left) and γδ T cells (right) in the photoconverted inguinal lymph node at the indicated time points after photoconversion. Correction is performed by subtracting the frequency of D-Red^{+} cells in the nonconverted inguinal lymph node from the frequency of D-Red^{+} cells in the converted inguinal lymph node for the respective population at each time point (n = 4 mice per time point in 3 independent experiments, mean±SD, one-way ANOVA with Tukey's multiple comparisons test, *: $P<0.05$; **: $P<0.01$; ***: $P<0.001$).

Supplementary Figure S5

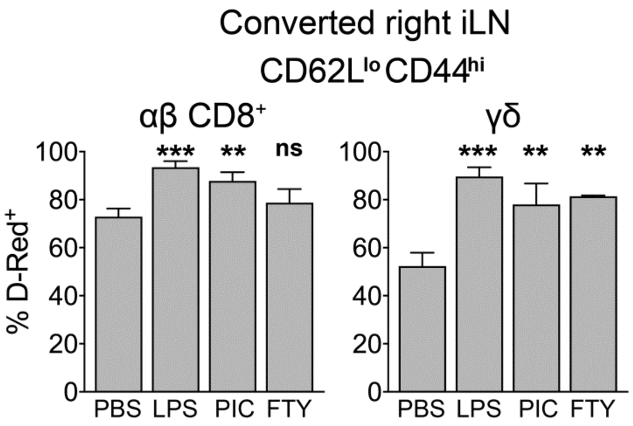

Figure S5 | Circulation of CD62L^{lo}CD44^{hi} αβ CD8^{+} and γδ T cells after systemic inflammation. Frequency of D-Red^{+} cells among CD62L^{lo}CD44^{hi} among αβ CD8^{+} T cells (left) and γδ T cells (right) in photoconverted iLNs (n = 3-5 mice per group in 4 independent experiments, mean±SD, one-way ANOVA with Tukey's multiple comparisons test, **: P<0.01; ***: P<0.001; ns: not significant). As described in Figure 3b-e, VHD mice are injected intraperitoneally with either PBS, LPS, poly(I:C) (PIC) or FTY720 (FTY) 2 hours after photoconversion of iLN and analyzed 16 hours after photoconversion.

Supplementary Figure S6

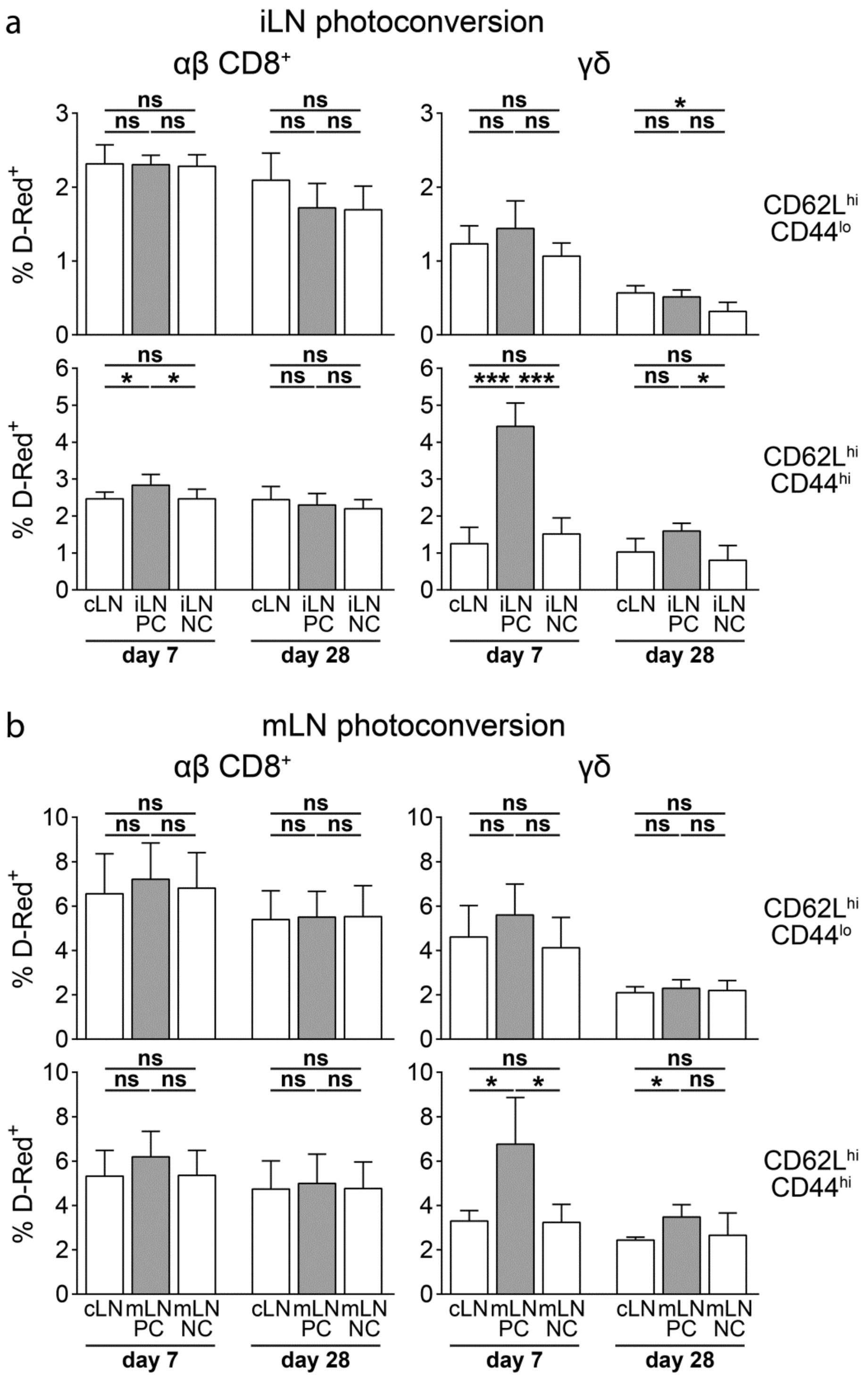

Figure S6 | Long term circulation kinetics of αβ CD8⁺ and γδ T cell subsets after photoconversion. (**a**) Frequency of D-Red⁺ cells among $CD62L^{hi}CD44^{lo}$ (top) and $CD62L^{hi}CD44^{hi}$ (bottom) for αβ CD8⁺ T cells (left) and γδ T cells (right) in the indicated LNs 7 and 28 days after photoconversion of iLN (n = 4-6 mice per time point in 5 independent experiments, mean±SD, one-way ANOVA with Tukey's multiple comparisons test, *: $P<0.05$; ***: $P<0.001$; ns: not significant). (**b**) Frequency of D-Red⁺ cells

among CD62L^{hi}CD44^{lo} (top) and CD62L^{hi}CD44^{hi} (bottom) for αβ CD8^{+} T cells (left) and γδ T cells (right) in the indicated LNs 7 and 28 days after photoconversion of mLN (n = 4-5 mice per time point in 5 independent experiments, mean±SD, one-way ANOVA with Tukey's multiple comparisons test, *: P<0.05; ns: not significant).
